# Relationship between plasma levels of zonulin, bacterial lipopolysaccharides, d-lactate and markers of inflammation in haemodialysis patients

**DOI:** 10.1007/s11255-016-1495-5

**Published:** 2017-01-02

**Authors:** Joanna Ficek, Katarzyna Wyskida, Rafał Ficek, Jarosław Wajda, Dariusz Klein, Joanna Witkowicz, Sylwia Rotkegel, Urszula Spiechowicz-Zatoń, Joanna Kocemba-Dyczek, Jarosław Ciepał, Andrzej Więcek, Magdalena Olszanecka-Glinianowicz, Jerzy Chudek

**Affiliations:** 1 Pathophysiology Unit, Department of Pathophysiology, School of Medicine in Katowice, Medical University of Silesia, Medyków 18 Street, 40-752 Katowice, Poland; 2 Health Promotion and Obesity Management Unit, Department of Pathophysiology, School of Medicine in Katowice, Medical University of Silesia, Katowice, Poland; 3 Department of Nephrology, Transplantation and Internal Medicine, School of Medicine in Katowice, Medical University of Silesia, Katowice, Poland; 4 Dialysis Center in Rybnik, Regional Specialist Hospital No. 3 in Rybnik, Rybnik, Poland; 5 Dialysis Center in Tychy, Centrum Dializa Sosnowiec, Tychy, Poland; 6 Dialysis Center in Pszczyna, Centrum Dializa Sosnowiec, Pszczyna, Poland; 7 Dialysis Center in Siemianowice Śląskie, Nefrolux, Siemianowice Śląskie, Poland; 8 Dialysis Center in Katowice, Centrum Dializa Sosnowiec, Katowice, Poland; 9 Dialysis Center in Chorzów, Centrum Dializa Sosnowiec, Chorzów, Poland; 10 Dialysis Center in Żory, Centrum Dializa Sosnowiec, Żory, Poland; 11 Dialysis Center in Wodzisław Śląski, Centrum Dializa Sosnowiec, Wodzisław Śląski, Poland; 12 Dialysis Center in Sosnowiec, Centrum Dializa Sosnowiec, Sosnowiec, Poland

**Keywords:** Zonulin, Chronic renal failure, Haemodialysis, d-Lactate, Inflammation, Intestinal permeability

## Abstract

**Background:**

Increased permeability of the intestinal wall and intestinal dysbiosis may contribute to chronic systemic inflammation, one of the causes of accelerated atherosclerosis and cardiovascular morbidity and mortality burden in patients with chronic kidney disease. The aim of this study was to evaluate the association between markers of intestinal permeability and inflammation in haemodialysis (HD) patients.

**Methods:**

Plasma concentration of zonulin, haptoglobin, TNFα, IL6, d-lactates and bacterial lipopolysaccharides (LPS) was assessed in blood samples obtained after overnight fast before midweek morning HD session in 150 stable, prevalent HD patients. Daily intake of energy and macronutrients was assessed on the basis of a food frequency questionnaire.

**Results:**

Serum hsCRP level was increased in over 70% of patients. Plasma levels of zonulin [11.6 (10.9–12.3) vs 6.8 (5.8–7.8) ng/mL], IL6 [6.2 (1.0–10.3) vs 1.3 (1.0–2.0) pg/mL] and TNFα [5.9 (2.9–11.8) vs 1.6 (1.3–1.8) pg/mL], but not LPS and d-lactates were significantly higher in HD than in healthy controls. d-lactates and LPS levels were weakly associated with IL6 (*R* = 0.175; *p* = 0.03, and *R* = 0.241; *p* = 0.003). There was a borderline correlation between plasma zonulin and serum hsCRP (*R* = 0.159; *p* = 0.07), but not with IL6, LPS and d-lactates. In multiple regression, both serum CRP and plasma IL6 variability were explained by LPS (*β* = 0.143; *p* = 0.08 and *β* = 0.171; *p* = 0.04, respectively), only.

**Conclusion:**

The weak association between plasma d-lactate, LPS and IL6 levels indicates that intestinal flora overgrowth or increased intestinal permeability contributes very slightly to the chronic inflammation development in HD patients.

## Introduction

The accelerated development of atherosclerosis is one of the main causes of precocious cardiovascular morbidity and mortality in dialysis patients with chronic kidney disease (CKD) [1]. It can be explained by the co-occurrence of classic cardiovascular risk factors (age, sex, hypertension, lipid disorders, diabetes or obesity) and the so-called uraemia-specific risk factors, which include CKD-MBD (chronic kidney disease—mineral and bone disorders), oxidative stress, accumulation of advanced glycation end products (AGEs), uraemic retention solutes (e.g. guanidine, asymmetric dimethylarginine (ADMA), p-cresol, indoxyl sulphate), as well as low-grade chronic systemic inflammation (microinflammation) [2–5].

The pathogenesis of chronic inflammation in haemodialysis (HD) patients is complex and not entirely explained. It is closely related to atherosclerosis by a bidirectional association. Inflammation accelerates the progress of atherosclerosis, and during atherogenesis development, an activation of monocytes and macrophages that secrete pro-inflammatory cytokines (e.g. TNFα) and chemokines occurs. In addition, visceral adipose tissue (the sources of pro-inflammatory cytokines) in patients with abdominal obesity, contact with blood by the dialyser membrane and probably increased intestinal permeability are contributing to increased inflammatory state [6].

During the last few years, the importance of intestinal microflora contributing to the development of autoimmune diseases, hormonal metabolism and systemic inflammation was raised [7, 8]. Impaired structure and function of the intestinal barrier related to dysbiosis may increase the penetration of bacteria-derived toxins (including lipopolysaccharides), or even viable bacteria into the blood. This mechanism can induce systemic inflammation [8, 9]. Accumulation of uraemic toxins and metabolic disorders in CKD patients may influence the composition of intestinal flora and promote gut permeability. That may lead to depletion of immune system mechanisms and finally to the state of immunodeficiency and chronic systemic inflammation; however, the data supporting this hypothesis are limited [6]. Wang et al. [10] found the evidence for intestinal flora translocation (bacterial ribosomal DNA in blood) in six CKD stage 5 patients. The presence of bacterial DNA was also associated with significantly higher levels of highly sensitive CRP, IL6 and d-lactates [11].

Recently zonulin, a new marker potentially useful for the evaluation of the permeability of intestinal wall, was described [12]. Zonulin is a protein (molecular weight of 47 kDa), HP2 gene product (which simultaneously encodes haptoglobin), which interacts with intercellular connections (*Zona occludens*) [13]. Increased concentration of zonulin was observed in patients with sepsis [14], obesity [7], type 2 diabetes [15] and autoimmune diseases [16]. It has not been established whether synthesis of zonulin is altered in CKD patients.

Additionally, measurement of d-lactate (a product of anaerobic metabolism from intestinal bacteria) concentration in the circulation may reflect colonic absorption of bacterial metabolism products. d-lactate and bacterial endotoxins are considered primarily as markers of colon absorption, partially reflecting the permeability of the intestinal wall [17, 18].

The aim of this study was to evaluate the association between markers of intestinal permeability and systemic inflammation in haemodialysis patients.

## Subjects and methods

**Fig. 1:**
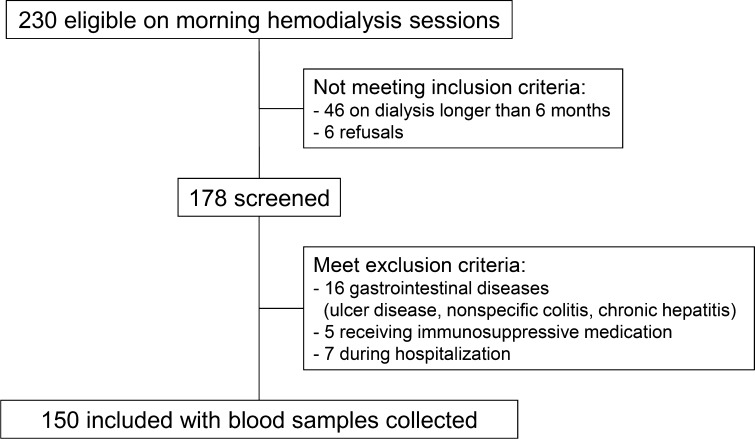
Study flow chart

One hundred and fifty (*N* = 150) stable, prevalent HD patients (92 men and 58 women) were enrolled into the study. Only patients on morning dialysis sessions were included due to requirement of morning blood samples collection (after overnight fast). Patients with previous history of gastrointestinal diseases, receiving immunosuppressive medication, during hospitalization and on HD therapy for less than 6 months were excluded (Fig. 1). The study protocol was accepted by the Bioethical Committee of Medical University of Silesia in Katowice (KNW-2-015/N/3/K), and each patient gave informed consent for participation in the study. The study protocol neither included training with a nutritionist nor interfered with previous nutrition recommendations.

All HD patients were dialysed 3 times per week for 3.5–5 h (11.7 ± 0.9 h weekly) using low-flux dialysers with polyethersulphone (PES) or polysulphone (PSU) membranes (area range 1.5–2.0 m^2^), with blood flow rates from 200 to 250 ml/min and dialysate flow rate 500 ml/min. Patient characteristics including CKD causes, duration of HD therapy and Kt/V are given in Table 1.

**Table 1:** Demographic and clinical characteristics of 150 haemodialysis patients and 30 control subjects (mean and 95% CI)

	Haemodialysis patients (*N* = 150)	Control subjects (*N* = 30)
Age (years)	62 (59–64)	53 (49–57)
Gender (male/female)	92/58	21/9
Body mass index (kg/m^2^)	26.1 (25.2–26.9)	26.5 (24.8–28.3)
Obesity (BMI ≥ 30 kg/m^2^) (*n*/ %)	28/18.7	9/30.0
Primary cause of CKD (*n*/ %)		
*Diabetes*	42/28.0	NA
*Hypertension*	17/11.3	NA
*Nephrolithiasis*	8/5.3	NA
*Autosomal dominant polycystic kidney disease (ADPKD)*	10/6.7	NA
*Ischaemic nephropathy*	2/1.3	NA
*Glomerulonephritis*	24/16.0	NA
*Interstitial nephritis*	13/8.5	NA
*Other or unknown*	34/22.7	NA
Time on dialysis (months)	48 (41–56)	NA
Kt/V (per HD session)*^*	1.05 (1.01–1.08)	NA
Urea reduction ratio (%)	64.8 (63.3–66.2)	NA
Comorbidity (%)		
*Hypertension*	136/90.7	0
*Diabetes*	55/36.7	0
*Coronary artery disease*	83/55.3	0
*Stroke*	12/8.0	0
*Past kidney transplantation*	11/7.3	0
Pharmacotherapy (*n*/%)		
*Antihypertensive*	136/90.7	0
*No. of antihypertensive drugs* (*n*)	2.0 (1.8–2.2)	0
*Oral anti*-*diabetic*	19/34.5*	0
*Insulin*	36/65.5*	0
*Anti-platelet*	77/51.3	0
*Statins*	60/40.0	0
*Fibrates*	0	0
*Oral phosphorous binders*	129/86.0	0
*Carbonate calcium dose* (g/day)	3.8 (3.4–4.3)	0
*Sevelamer hydrochloride*	4/2.6	0
*Cinacalcet*	17/11.3	0
*Cinacalcet dose* (mg/day)	79 (60–98)	
*Alfacalcidol*	31/20.7	0

*NA* not applicable

* Percentage of patients with diabetes

^ Mean value from last 6 months

Thirty subjects (9 women and 21 men) without concomitant diseases: 9 obese and 21 normal weight, served as a control group. The exclusion criteria included acute or chronic diseases, any drug use, including oral contraceptive agents, body mass changes exceeding more than 3 kg during preceding 6 months, cigarette smoking, drinking more than three drinks per week, endocrine disorders: hyper- and hypothyroidism, Cushing’s syndrome, polycystic ovary syndrome.

The study protocol assumed a single withdrawal of an additional blood sample, while performing routine tests (blood count, serum urea, calcium, phosphate, sodium, potassium), before the midweek HD session after overnight fasting. Only patients on morning haemodialysis sessions were recruited. Also in the control group, blood samples were obtained in the morning after overnight fast.

### Measurements

Plasma concentrations of zonulin (Immundiagnostik AG, Bensheim, Germany) haptoglobin (AssayPro, Saint Charles, Mo, U.S.), TNFα and IL6 (R&D Systems, Minnesota, MN, U.S.), d-lactates (Uscn Life Sciences Inc., Wuhan, PRC) and bacterial lipopolysaccharides (Uscn Life Sciences Inc., Wuhan, PRC) were assessed by ELISA using commercially available kits. The intra-assay and inter-assay coefficients of variability were as follows: <5 and <8.5% (zonulin), <4.9 and <7.5% (haptoglobin), <7.3 and <4.3% (TNFα), <7.2 and <7.8% (IL6), <8 and <10% (d-lactates), <10 and <12% (lipopolysaccharides).

Serum hsCRP was assessed by immunoturbidimetric method (DRG Instruments GmbH for Hybrid XL, Marburg, Germany) with inter-assay precision <4.8%.

### Statistical analysis

Statistical analysis was performed with STATISTICA 10.0 PL StatSoft, Inc. software (www.statsoft.com). The normality of quantitative variables distribution was checked by Shapiro–Wilk test. Results are given as mean values with standard deviation or 95% confidence intervals (95% CI) or medians with interquartile range (variables with skewed distribution). Study population was divided in tertiles based on plasma concentration of zonulin, lipopolysaccharides and d-lactates. For comparison of groups (HD patients and controls, and subgroups), we used the Chi-square test (qualitative variables), and ANOVA or Kruskal–Wallis one-way analysis of variance by ranks followed by Mann–Whitney *U* test (quantitative variables). Correlation coefficient was calculated according to Spearman.

Value of *p* < 0.05 was considered statistically significant in all analyses.

## Results

### Study group characteristics

One hundred and fifty haemodialysis patients were included in the study (more than two-thirds of them were men). The mean duration of dialysis time was 4 years. Average BMI was 26 kg/m^2^, and 18 (12%) patients met the criteria for obesity. Causes of CKD were typical; the most common cause was diabetes, then glomerulonephritis and finally, hypertensive nephropathy (Table 1). The majority of patients were suffering from arterial hypertension, more than half of them from coronary disease, and one-third from diabetes. Dozens of patients having prior stroke and a similar group had a history of past kidney transplantation. Half of the patients took anti-platelet agents and over one-third statins. Oral phosphorous binders were prescribed for the majority of the patients (*n* = 129) (Table 1).

**Fig. 2:**
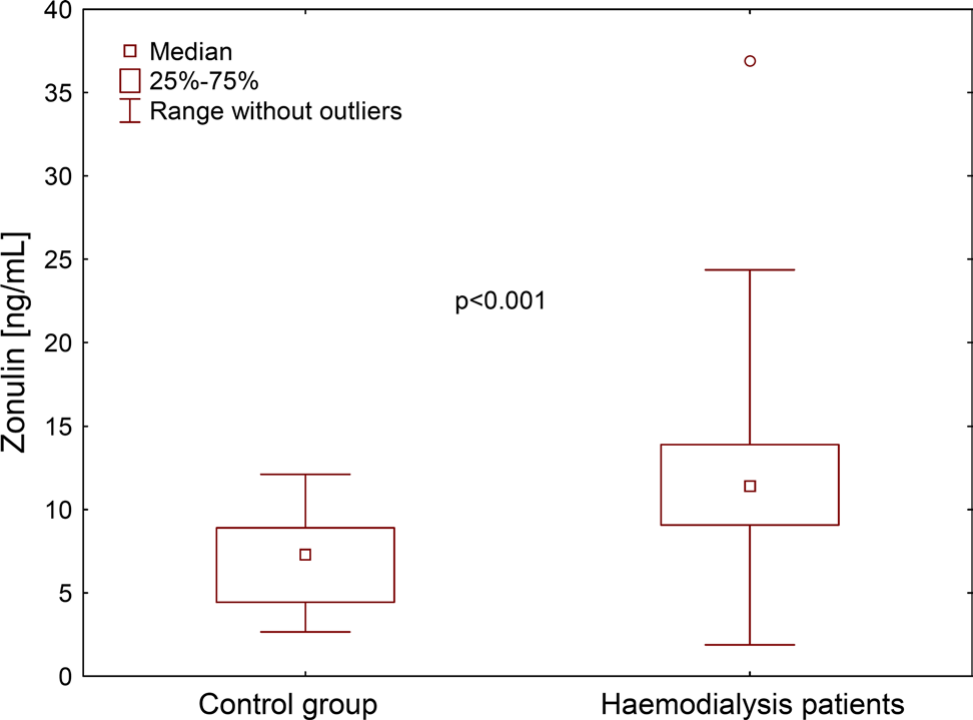
Plasma zonulin concentration in 150 haemodialysis patients and 30 control subjects

The average value of haemoglobin (10.8 g/dL) suggested effective treatment for patients with anaemia. The mean serum concentration of total and LDL cholesterol was within the normal range, while the average serum HDL concentration was low. Triglyceride concentration exceeded the upper limit; typical for CKD stage 5 calcium–phosphate disturbances with secondary hyperparathyroidism were observed. Inflammatory markers (serum hsCRP and plasma TNFα) were slightly elevated (Table 2). An increased hsCRP level (over 3 mg/dL) was found in over 70% of patients. The levels of zonulin (Fig. 2), hsCRP, IL6 and TNFα were significantly increased in relation to values in control subjects (Table 2). The levels of haptoglobin, LPS and d-lactates were similar in HD and controls.

**Table 2:** Biochemical characteristics and the study parameters

	Haemodialysis patients [*N*=	Control subjects [*N* = 30]	*p*
Haemoglobin (g/dL)*^*	10.8 (10.6–11.0)	13.8 (11.2–14.9)	<0.01
Total cholesterol (mg/dL)	169 (160–178)	198 (183–214)	<0.05
LDL cholesterol (mg/dL)	90 (84–95)	131 (117–146)	<0.05
HDL cholesterol (mg/dL)	28 (26–29)	57 (50–64)	<0.01
Triglycerides (mg/dL)	169 (160–178)	114 (95–133)	<0.05
Calcium (mg/dL)*^*	8.57 (8.44–8.70)	ND	–
Phosphorous (mg/dL)*^*	5.77 (5.52–6.02)	ND	–
Parathyroid hormone (pg/mL)	449 (380–519)	ND	–
hsCRP (mg/L)*	4.82 (2.33–11.60)	1.71 (0.87–3.19)	<0.05
Interleukin 6 (pg/mL)*	6.20 (1.01–10.32)	1.34 (0.99–1.97)	<0.05
Tumour necrosis factor-α (pg/mL)*	5.94 (2.93–11.80)	1.61 (1.31–1.85)	<0.01
Bacterial lipopolysaccharides (ng/mL)	27.7 (22.1–36.9)	28.1 (16.6–57.6)	0.72
d-lactates (µg/mL)*	1.47 (0.94–2.34)	1.55 (1.35–1.77)	0.63
Zonulin (ng/mL)	11.6 (10.9–12.3)	6.8 (5.8–7.8)	<0.01
Haptoglobin (µg/mL)*	1.37 (0.82–2.03)	1.39 (0.98–1.72)	0.87

*ND* not determined

Mean and 95% CI or * Median (25–75 percentile)

^ Mean value from last 6 months

Zonulin level variability was not explained by residual diuresis, Kt/V and urea reduction ratio.

### Subgroups analysis

**Fig. 3:**
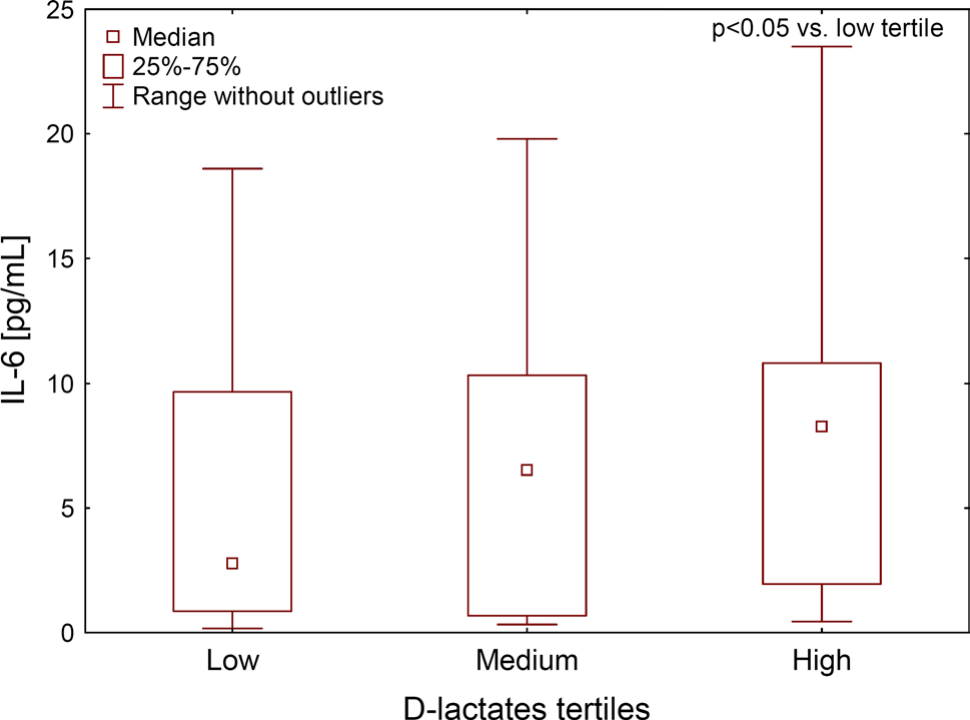
Plasma interleukin 6 concentration in subsequent d-lactates concentration tertiles

The zonulin tertiles subgroups of HD patients differ in respect of hsCRP and TNFα, but had similar levels of d-lactates, LPS and haptoglobin. Similar analysis for LPS tertiles showed increasing IL6 levels in the subsequent tertiles (Fig. 3). In addition, d-lactates tertiles differed in respect of IL6 and haptoglobin, but not LPS and zonulin levels (Table 3).

**Table 3:** Biochemical characteristics and the study parameters in tertile subgroups of zonulin, bacterial lipopolysaccharides and d-lactates

	Zonulin (ng/mL)			ANOVA/ Kruskal–Wallis
	Low tertile <9.8	Medium tertile	High tertile >13.1	
Haemoglobin (g/dL)	10.6 (10.3–10.9)	10.8 (10.5–11.2)	10.9 (10.5–11.3)	*p* = 0.43
Total cholesterol (mg/dL)	169 (155–183)	161 (146–176)	177 (161–193)	*p* = 0.32
LDL cholesterol (mg/dL)	89 (80–99)	85 (75–94)	95 (84–106)	*p* = 0.35
HDL cholesterol (mg/dL)	28 (26–30)	28 (26–31)	27 (24–30)	*p* = 0.72
Triglycerides (mg/dL)	158 (134–182)	158 (120–196)	162 (134–190)	*p* = 0.97
hsCRP (mg/L)*	3.96 (1.58–10.32)	4.04 (2.20–8.78)	5.76 (3.58–13.20)^	*p* = 0.07
Interleukin 6 (pg/mL)*	6.39 (1.01–9.66)	4.08 (0.78–9.70)	8.35 (1.28–11.31)	*p* = 0.12
Tumour necrosis factor-α (pg/mL)*	4.41 (2.36–8.26)	7.07 (3.12–15.60)^	7.01 (3.16–11.80)^	*p* = 0.04
Bacterial lipopolysaccharides (ng/mL)*	26.9 (22.2–37.0)	28.5 (21.8–35.6)	27.0 (22.9–37.3)	*p* = 0.98
d-Lactates (µg/mL)*	1.77 (0.96–2.62)	1.29 (0.91–2.21)	1.42 (0.98–2.30)	*p* = 0.23
Haptoglobin (µg/mL)*	1.37 (0.74–1.91)	1.27 (0.84–2.36)	1.64 (0.91–2.30)	*p* = 0.54

	Bacterial lipopolysaccharides (ng/mL)			
	Low tertile <24.1	Medium tertile	High tertile >32.6	
Haemoglobin (g/dL)	10.6 (10.3–10.9)	10.9 (10.6–11.2)	10.9 (10.4–11.3)	*p* = 0.47
Total cholesterol (mg/dL)	171 (153–188)	176 (163–191)	159 (146–173)	*p* = 0.26
LDL cholesterol (mg/dL)	87 (76–97)	95 (86–104)	87 (77–98)	*p* = 0.41
HDL cholesterol (mg/dL)	27 (25–29)	29 (26–31)	27 (25–30)	*p* = 0.65
Triglycerides (mg/dL)	178 (137–219)	153 (129–177)	146 (123–170)	*p* = 0.29
hsCRP (mg/L)*	3.73 (1.80–11.04)	4.84 (3.42–9.08)	5.48 (2.58–15.92)	*p* = 0.21
Interleukin 6 (pg/mL)	3.48 (0.78–8.38)	6.29 (1.20–9.52)	9.11 (1.09–11.80)^#^	*p* = 0.02
Tumour necrosis factor-α (pg/mL)*	4.94 (2.31–10.10)	6.46 (3.06–12.30)	8.26 (3.15–12.90)	*p* = 0.35
d-Lactates (µg/mL)*	1.65 (0.97–2.38)	1.33 (0.95–2.34)	1.47 (0.94–2.21)	*p* = 0.70
Haptoglobin (µg/mL)*	1.43 (0.84–2.36)	1.33 (0.83–2.30)	1.37 (0.73–1.92)	*p* = 0.63
Zonulin (ng/mL)	10.8 (9.7–11.9)	12.2 (11.2–13.2)	11.8 (10.1–13.4)	*p* = 0.24

	d-Lactates (µg/mL)			
	Low tertile <1.14	Medium tertile	High tertile >2.0	
Haemoglobin (g/dL)	10.7 (10.4–11.1)	10.7 (10.4–11.1)	10.9 (10.5–11.2)	*p* = 0.80
Total cholesterol (mg/dL)	165 (153–177)	170 (153–186)	173 (156–189)	*p* = 0.76
LDL cholesterol (mg/dL)	89 (80–98)	85 (76–95)	94 (83–106)	*p* = 0.44
HDL cholesterol (mg/dL)	28 (25–30)	28 (25–30)	27 (25–30)	*p* = 0.98
Triglycerides (mg/dL)	150 (126–174)	172 (137–208)	156 (124–187)	*p* = 0.57
hsCRP (mg/L)*	4.84 (2.58–12.60)	5.20 (2.61–10.32)	3.87 (2.15–11.04)	*p* = 0.70
Interleukin 6 (pg/mL)*	2.78 (0.86–9.66)	6.54 (0.68–10.33)	8.27 (1.95–10.80)^	*p* = 0.04
Tumour necrosis factor-α (pg/mL)*	6.33 (3.15–15.63)	7.07 (2.48–12.20)	4.68 (2.59–10.20)	*p* = 0.15
Bacterial lipopolysaccharides (ng/mL)*	27.5 (21.4–37.6)	27.7 (23.3–36.6)	28.0 (21.3–34.4)	*p* = 0.88
Haptoglobin (µg/mL)*	1.00 (0.71–1.73)	1.64 (1.03–2.33)^	1.39 (0.89–2.38)^	*p* = 0.02
Zonulin (ng/mL)	11.4 (10.4–12.3)	12.7 (11.1–14.4)	10.7 (9.6–11.8)	*p* = 0.12

Mean and 95% CI or * median (25–75 percentile)

^* p *  < 0.05; ^#^ *p* < 0.01 versus first tertile

### Correlations between LPS, d-lactates and zonulin, and inflammatory markers

**Fig. 4:**
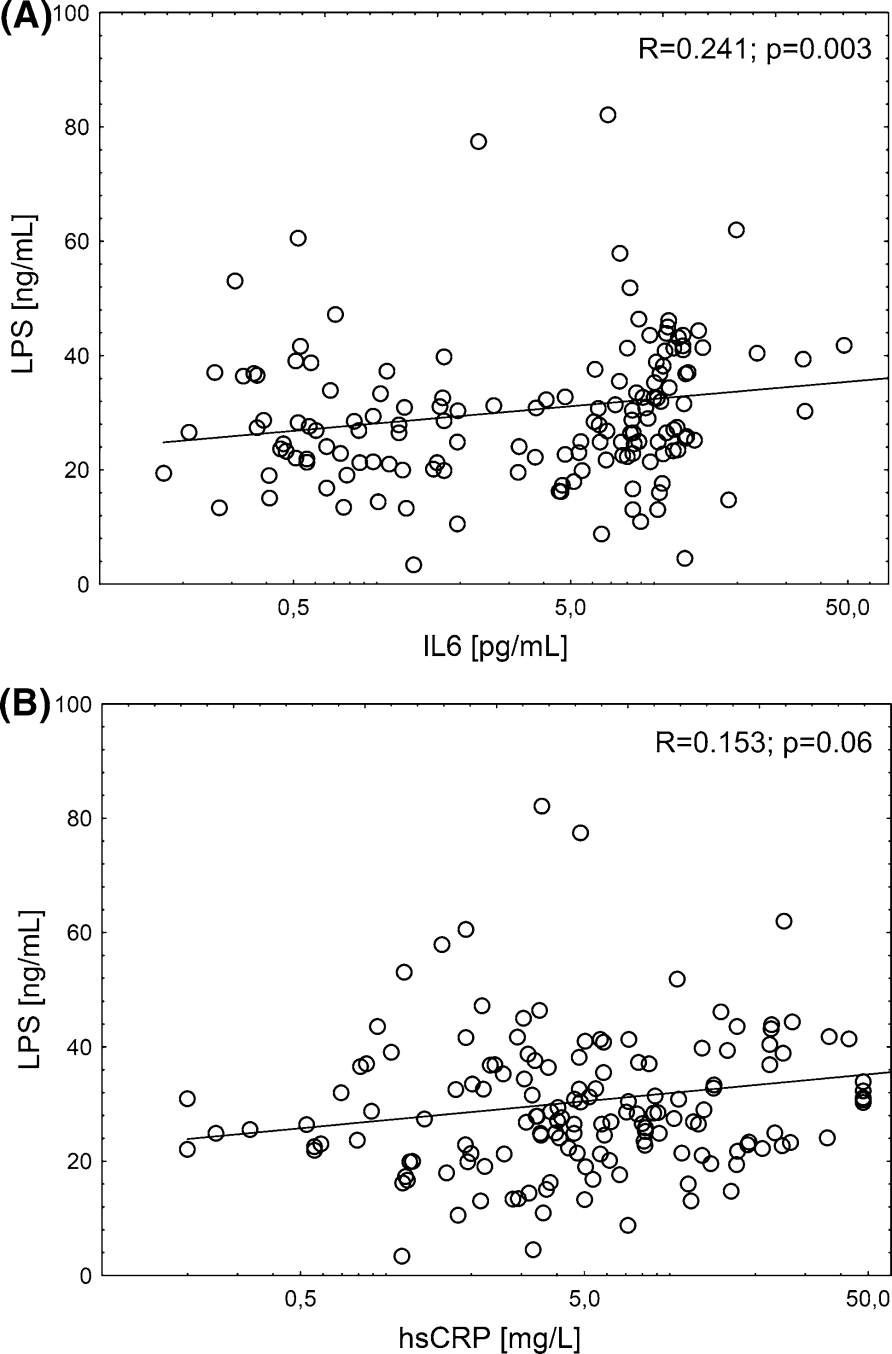
Correlations between plasma concentration of LPS and inflammatory markers: **a** IL6 (*R* = 0.241; *p* = 0.003) and **b** serum hsCRP level (*R* = 0.153; *p* = 0.06)

There was a stronger correlation between plasma concentrations of LPS and IL6 (*R* = 0.241; *p* = 0.003) than with serum hsCRP level (*R* = 0.153; *p* = 0.06) (Fig. 4). No association with TNFα was found (*R* = 0.101; *p* = 0.22). The levels of d-lactates were weakly associated with IL6 (*R* = 0.175; *p* = 0.03), but neither with hsCRP (*R* = −0.022; *p* = 0.78) nor with TNFα (*R* = −0.139; *p* = 0.09).

There was a borderline correlation between plasma zonulin and serum hsCRP (*R* = 0.159; *p* = 0.07). No correlation with plasma IL6 (*R* = 0.118; *p* = 0.15), TNFα (*R* = 0.114; *p* = 0.17), LPS (*R* = 0.019; *p* = 0.82) and d-lactates (*R* = −0.083; *p* = 0.31) was found.

The plasma levels of haptoglobin were weakly related to those of zonulin (*R* = 0.138; *p* = 0.09) and d-lactates (*R* = 0.186; *p* = 0.02).

### Multiple regression models

In multiple regression models including plasma concentrations of zonulin, LPS and d-lactates, both serum CRP and plasma IL6, but not plasma TNFα variability was explained only by LPS (*β* = 0.143; *p* = 0.08 and *β* = 0.171; *p* = 0.04, respectively).

## Discussion

The levels of zonulin, hsCRP, IL6 and TNFα were significantly higher in HD patients, while the levels of haptoglobin, LPS and d-lactates were similar in both study groups. Additionally, a weak association between plasma levels of IL6 and d-lactate as well as LPS were observed, while in multiple regression models, serum hsCRP and plasma IL6 variability were explained by LPS, only.

Moderate increase of circulating inflammatory markers (CRP, IL6 and TNFα) is typical for CKD patients [4, 5]. In addition, the relationship between systemic inflammation in CKD patients and the severity of endotoxaemia was reported [19]. Moreover, in animals with experimentally induced CKD, a bacterial DNA was detected in blood, spleen, lymph nodes and the liver [20]. The most likely source of bacteria is the gut microflora. In CKD, impaired intestinal epithelium cellular tight junctions, which allow the passage of endotoxins and bacteria into circulation, were observed [21, 22]. In addition, an altered composition of the intestinal microflora of CKD patients was described. This change can be explained by uraemia and additionally by specific diet and medications used in those patients [10]. However, in our study despite higher zonulin levels in the circulation of HD patients, suggesting increased gut permeability, similar concentrations of LPS and d-lactates were found. In addition, similar concentrations of LPS were showed in zonulin tertiles. These data do not confirm significant endotoxaemia in the majority of HD patients without gastrointestinal diseases and raise the uncertainty concerning zonulin as the marker of gut permeability in HD. It should be noted that until now, plasma zonulin (prehaptoglobin-2) concentration has not been evaluated in HD patients. Our study shows elevated plasma levels of zonulin in these patients even after comparison with obese subjects [8.2 (7.1–8.4) ng/mL] [7]. The cause of increased zonulin level in HD is unclear, also taking into account the lack of elevation of plasma haptoglobin (encoded by the same gene as zonulin). Perhaps increased plasma zonulin levels in HD result from decreased degradation/elimination by the kidneys.

One of the examined parameters was plasma level of d-lactic acid, a surrogate marker of colon bacteria overgrowth. d-lactic acid exists as two stereoisomers: right-handed (d) and left-handed (l). Both isomers, as a result of the action of a specific lactate dehydrogenase (l and d) (via pyruvate conversion), d- or l-lactic acid molecule is produced. In human cells, activity of d-lactate dehydrogenase is not observed, and naturally, only the l-form of lactic acid occurs. However, d-lactic acid can be present in the blood stream and reflect exogenous origin or be the result of methylglyoxal metabolism. Methylglyoxal may be derived from carbohydrates, fatty acid or amino acids transformation. It is a toxic compound and therefore, via glyoxalase 1 and glyoxalase 2, is converted to d-lactate. Furthermore, d-lactates can be derived from enteric bacterial fermentation predominantly of *Lactobacillus* and *Bifidobacteria* [23].

Increased plasma concentration of d-lactates may occur in the case of sugar overabundance in the diet, short bowel syndrome, as well as microbial proliferation and excessive fermentation [23]. It is not clear whether increased intestinal permeability can be accompanied by progression of kidney disease or can be due to influence of uraemic toxins on the intestinal wall. Some studies indicate the role of urea in this process, but it does not seem to be a key element in the development of chronic microinflammation. Considering the role of intestinal microflora in the development of autoimmune diseases and systemic inflammation shown in clinical trials [7], we design this study to explain whether chronic inflammation in HD patients corresponds to increased intestinal permeability and uraemia-related dysbiosis. However, in our study, plasma levels of d-lactates were similar in HD and controls and were weakly associated with plasma IL6 concentration. Thus, it seems that microbial proliferation and excessive fermentation in the colon are only mild factors participating in the systemic microinflammation in HD patients.

A weak correlation between levels of IL6 and d-lactates and LPS, and the greatest severity of inflammation were found in the highest tertile of zonulin, LPS and d-lactates concentrations, while in multiple regression models, serum hsCRP and plasma IL6 variability were explained by LPS, only. This supports the hypothesis that bacteria overgrowth in the gut or the increased permeability of intestinal wall only in a small part participates to the inflammation development in HD patients. In addition, the weakness of these relationships confirms that the other well-known mechanisms of systemic inflammation in HD [2] have a predominant role.

The study has some limitations, including the lack of the intestinal microflora analysis and the absence of microflora bacterial (ribosomal) DNA identification in plasma samples, and the lack of performance of intestinal permeability tests. Furthermore, the cross-sectional study design precludes the analysis of cause-and-effect relations.

## Conclusion

The weak association between plasma d-lactate, LPS and IL6 levels indicates that intestinal flora overgrowth or increased intestinal permeability contributes very slightly to the chronic inflammation development in HD patients.
